# An Energetic Tradeoff Best Explains Parturition Timing in Grizzly Bears

**DOI:** 10.1002/ece3.72914

**Published:** 2026-01-21

**Authors:** Cecily M. Costello, Lori L. Roberts, Daniel D. Bjornlie, Matthew D. Cameron, Justin G. Clapp, Mark A. Haroldson, Grant V. Hilderbrand, Kyle Joly, Wayne F. Kasworm, Jeremy M. Nicholson, Thomas G. Radandt, Mathew S. Sorum, Justin E. Teisberg, Frank T. van Manen, Milan A. Vinks

**Affiliations:** ^1^ Montana Fish, Wildlife and Parks Kalispell Montana USA; ^2^ Wyoming Game and Fish Department Lander Wyoming USA; ^3^ U.S. Fish and Wildlife Service Anchorage Alaska USA; ^4^ Gates of the Arctic National Park and Preserve, National Park Service Fairbanks Alaska USA; ^5^ Northern Rocky Mountain Science Center Interagency Grizzly Bear Study Team, U.S. Geological Survey Bozeman Montana USA; ^6^ National Park Service Alaska USA; ^7^ U.S. Fish and Wildlife Service Libby Montana USA; ^8^ Idaho Fish and Game Department Idaho Falls Idaho USA

**Keywords:** birth, body condition, brown bear, cub survival, delayed implantation, denning chronology, hibernation, reproduction, *Ursus arctos*

## Abstract

Timing of grizzly bear ( *Ursus arctos* ) parturition during hibernation has been explained by ancestral traits (delayed implantation, altricial young, obligate maternal denning), but the ultimate driver underlying precise timing has not been fully explored. Capitalizing on an observed latitudinal increase in denning duration among four populations in interior North America, we tested two alternative hypotheses. First, that birth timing results from a physiological cue that synchronizes implantation with the onset of hibernation, allowing females to forgo reproduction should they lack adequate fat stores. Alternatively, that parturition is optimally timed relative to den exit to balance an energetic tradeoff between minimizing lactation time to protect the mother and maximizing developmental time to increase cub survival. Using parturition dates previously predicted from accelerometer data (27 Dec–28 Feb), we classified 115 females according to apparent litter survival when first visually observed after den exit: 57% successful (with cubs), 22% unsuccessful (alone), and 21% unknown (not observed). The number of days between birth and den exit showed no association with latitude (*p* = 0.29). It averaged 103 days among successful females but only 77 days among unsuccessful females (*p* < 0.001) owing to later births and earlier exit. With each increasing degree of latitude, birth date increased by 1.0 and number of days between den entry and birth increased by 2.5 (*p* < 0.001). Implantation dates were not centered on den entry dates (*p* < 0.001). These results supported the energetic tradeoff hypothesis and suggested natural selection has favored a consistent number of days between parturition and den exit under average body conditions and shifts toward later or earlier births for females with lower or higher levels of bodily stored energy, respectively. This flexible tradeoff may support resilience to climate change and present a possible mechanism explaining reduced natality and cub survival in high‐density populations.

## Graphical abstract

Capitalizing on latitudinal denning‐chronology variation among four grizzly bear populations in interior North America, and using grizzly bear parturition dates (27 Dec–28 Feb) previously predicted from anomalous daily motion counts, we found evidence supporting the hypothesis that birth is optimally timed relative to den exit to balance an energetic tradeoff between minimizing lactation time to protect the mother and maximizing developmental time to increase cub survival.

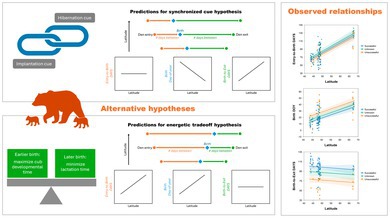

## 1 Introduction

Arising during the Pleistocene glaciations, most extant members of the Ursidae display distinct seasonality of births, which likely evolved along with three early ancestral traits: delayed implantation, highly altricial neonates, and obligate maternal denning behavior (Spady et al. 2007; Fowler et al. 2021). In contrast to some bear species that seek maternal dens simply for birthing and early cub rearing, brown bears and other members of the Ursinae subfamily give birth during winter hibernation, a refined denning behavior accompanied by physiological functions designed to reduce energy expenditure during this period of food shortage (Fowler et al. 2021). All pregnant females in these species give birth while hibernating, even in circumstances when conspecifics remain active to exploit available foods (e.g., Hellgren and Vaughan 1987; Van Daele et al. 1990; Amstrup 2003). Whereas this evolutionary history helps explain the general timing of bear births during winter, few studies have examined proximate causes (Friebe et al. 2014; Lemière et al. 2022), and none have explained the ultimate cause for precise timing of birth within a hibernation period that can last 4–8 months (Haroldson et al. 2021, Krofel et al. 2017, this study). In brown bears, births have generally been reported to occur during Jan–Feb, except among captive bears in the southern hemisphere where births are shifted by 6 months (e.g., Fowler et al. 2021).

The timing of birth within the hibernation period means the energetic costs of gestation and early lactation are borne by females using only bodily stored energy. Besides expending fat reserves for this reproductive effort and their own maintenance, parturient females must also use lean body mass to provide for the protein needs of their young and often as an energy source (Harlow et al. 2002; López‐Alfaro et al. 2013). This suggests at least a small reproductive impact on survival of adult females, as has been observed in Weddell seals ( *Leptonychotes weddellii* ; Hadley et al. 2007). In response to reproductive costs, long‐lived, iteroparous species often evolve energetic “prudent parent” strategies that prioritize maternal survival and future reproductive success over any single reproductive event (e.g., Drent and Daan 1980; Morano et al. 2013). In brown bears, such strategies should include optimizing the timing of parturition within the hibernation period to maximize lifetime fitness.

Using triaxial accelerometer data obtained from radio‐collars, summarized as time‐series of daily counts of activity readings > 0 measured at 10‐min intervals, Roberts et al. (2026) developed and evaluated a technique to predict occurrence and timing of births by detection of brief anomalous upsurges in activity likely associated with postnatal maternal behaviors. By applying the method to blind samples, they found the technique was 91% accurate in predicting parturition for females observed with cubs‐of‐the‐year (hereafter cubs) and 83% accurate in predicting no parturition event for females subsequently observed with older offspring. Roberts et al. (2026) and our study involved four interior North American brown bear (hereafter grizzly bear [Paetkau et al. 1998]) populations at varying latitudes, providing us with a large sample of predicted‐parturient females to evaluate parturition timing. Several studies have shown that denning duration is negatively correlated with growing season and positively associated with latitude (Ferguson and McLoughlin 2000; Haroldson et al. 2002; Krofel et al. 2017; González‐Bernardo et al. 2020). Capitalizing on this latitudinal trend in denning dates among our sample, we developed and tested two alternative hypotheses for the drivers of birth timing.

Our first hypothesis postulates the timing of parturition is governed by the timing of den entry due to physiological cues synchronizing implantation with the onset of hibernation (synchronized cue hypothesis). This birth timing would facilitate an “all‐or‐nothing” energetic strategy whereby current reproductive costs could be eliminated should their impact on maternal survival be high, allowing all stored energy to be allocated to future reproduction. At the start of hibernation, the stored energy (i.e., fat and lean mass) a female would need for maintenance, gestation, and lactation would be at its maximum. It follows that a proximate mechanism which temporally couples the start of gestation with this energy maximum would be adaptive if females were capable of forgoing implantation and pregnancy should they lack adequate energy, as has been postulated but not verified (Rogers 1976; Bunnell and Tait 1981; Robbins et al. 2012). Ultimately, this mechanism would function to eliminate unnecessary energetic costs of a likely‐to‐fail reproductive effort early in the hibernation period, thus maximizing energy savings. Several lines of evidence from studies of brown and American black bears ( *Ursus americanus* ) form a foundation for this hypothesis. First, apparent absence of litter production has been observed among females in poorer body condition or following years of low natural food availability (e.g., Costello et al. 2003, Robbins et al. 2012, McLellan 2015), leading to the supposition that females in poorer condition may forgo implantation. Second, earlier dates of den entry by pregnant females have been consistently observed in the wild and in captivity (Servheen and Klaver 1983; Judd et al. 1986; Schoen et al. 1987; Van Daele et al. 1990; Linnell et al. 2000; Friebe et al. 2001; Haroldson et al. 2002; Pigeon et al. 2016). In a long‐term study of captive wild‐caught American black bears, Mesa Cruz (2018) observed that pregnant females entered the physiological state of hibernation by voluntarily stopping their own food consumption, whereas nonpregnant females entered hibernation only when food was removed. Citing reports that blastocysts were not implanted until the female was in her den (Tsubota et al. 1990; Hissa 1997), Friebe et al. (2001) suggested that biochemical changes in blood parameters in preparation for hibernation (Hissa et al. 1994) may provide a physiological explanation for the early denning of pregnant females. Third, Mesa Cruz (2018) reported a close temporal relationship between the onset of hibernation and the earliest diagnosis of pregnancy via ultrasound. Their findings suggested possible physiological signals associated with implantation (e.g., cytokines or proteins) might also act as cues for seeking a den and hibernating. Earlier researchers argued that hibernation metabolism and reproductive physiology were not directly linked, based on the temporal separation of the progesterone spike assumed to be associated with implantation and the onset of denning behavior (Hellgren 1988, Palmer et al. 1988). Recent studies, however, have shown that giant pandas ( *Ailuropoda melanoleuca* ) might experience implantation up to 40 days after the observed rise in progesterone (Kersey et al. 2010, 2016), potentially explaining the mismatch. Under the hypothesis that implantation is synchronized with the onset of hibernation, we predicted the period between den entry and birth would be invariant among populations, leading to earlier births and a longer postpartum denning periods at higher latitudes due to longer denning duration.

Our second hypothesis proposes that parturition is ultimately timed in relation to den exit, spring green‐up, and resumption of maternal foraging (energetic tradeoff hypothesis). Optimizing the timing of birth to coincide with peak resource availability is a primary driver of seasonal breeding strategies (Bronson 1989). Much like the dichotomy influencing optimal hatching time in birds (e.g., Partridge 1989; Drent 2006), this energetic strategy for timing birth to maximize lifetime fitness involves optimizing a tradeoff between the energetic welfare of the mother and the survival potential of her current offspring. Lactation is more energetically demanding than gestation (López‐Alfaro et al. 2013), and the small size of ursid neonates is thought to be an evolutionary strategy to minimize costs of lactation during a period when mothers are either fasting or hibernating in a maternal den (Spady et al. 2007). Using energetic modeling, López‐Alfaro et al. (2013) estimated that increasing preemergence lactation time by only 2 weeks, from 60 to 74 days, would account for an additional 10% loss of lean body mass for females with a litter size of two cubs. Thus, minimizing the duration of lactation prior to the resumption of food intake would be advantageous for parturient females (Robbins et al. 2012; Lemière et al. 2022) and may impact their lifetime fitness. Conversely, maximizing the pre‐emergence developmental period for neonates also provides fitness benefits (Robbins et al. 2012, Lemière et al. 2022). Presumably, mortality risks from predation and other extrinsic causes increase dramatically outside of the den for small, altricial ursid young (Garrison et al. 2007; Gardner et al. 2014). Furthermore, many of the foods available in early spring are scattered, low‐energy grasses and herbs requiring continual grazing movements by mothers (e.g., Mattson et al. 1991; Costello et al. 2016). Cubs that have attained larger body size by den exit and the physical attributes needed for travel and escape (e.g., climbing ability) likely survive at higher rates. Thus, under the hypothesis that natural selection would balance this energetic tradeoff, we predicted the period between birth and den exit would be invariant among populations at different latitudes, leading to later births and longer prenatal denning periods at higher latitudes. This strategy would necessitate a decoupling of hibernation and implantation cues, making this hypothesis an alternative to the synchronized cue hypothesis.

## 2 Methods

### 2.1 Study Areas

This study was conducted using data obtained from grizzly bears in four interior study areas of western North America: Greater Yellowstone Ecosystem (GYE; 2013–2022) in Wyoming, Montana, and Idaho; Northern Continental Divide Ecosystem (NCDE; 2011–2021) in Montana; Selkirk and Cabinet‐Yaak Ecosystems (SCYE; 2012–2018) in Idaho, Montana, and British Columbia; and Gates of the Arctic National Park and Preserve (GAAR; 2014–2017) in north‐central Alaska (Costello et al. 2025). The GYE, NCDE, and SCYE study areas were in relatively remote areas of the central Rocky Mountains and were characterized by rugged terrain ranging from < 1000 m to > 3500 m elevation. Vegetation consisted of mixed‐grass prairies and riparian hardwood forests at lower elevations; conifer (*Pinus*, *Pseudotsuga*, *Abies*, and *Picea* spp.) forests interspersed with grasslands or sagebrush (*Artemesia* spp.) shrublands at mid elevations; and herbaceous alpine communities at the highest elevations. Climate was characterized by long cold winters and short cool summers, with most precipitation occurring as snow. The GAAR study area was in the largely roadless Brooks Range of northcentral Alaska with mountains > 2500 m in elevation. Situated above the Arctic Circle, vegetation consisted primarily of arctic and alpine tundra, with alder (*Alnus* spp.) and willow (*Salix* spp.) shrublands at lower elevations. Black spruce ( *Picea mariana* ) and birch ( *Betula papyrifera* ) woodlands occurred at lower elevations south of the range. Climate was defined by long and extremely cold winters, with snow depths exceeding 60 cm.

### 2.2 Field Methods

Bears were captured for research or management purposes and were fitted with GPS collars (Telonics Inc. Mesa, AZ) that collected and stored activity readings at 5‐ or 10‐min intervals via a tri‐axial accelerometer. Grizzly bear captures were conducted following protocols approved by Animal Care and Use Committees of Montana Fish, Wildlife & Parks (FWP09‐2017), University of Montana (052‐23HCCFC‐100623), U.S. Geological Survey (2012‐01), and National Park Service (AKR_GAAR_Gustine_GrizzlyBear_2014). The U.S. Fish and Wildlife Service issued capture permits for the lower‐48 grizzly bear populations listed as Threatened under the U.S. Endangered Species Act (USFWS 4[d] rule, 50 CFR 17.40[b]). In all study areas, we conducted observation flights in early spring to ascertain the reproductive status of each adult female, including age of offspring (i.e., cubs, yearlings, or 2‐year‐olds) and litter size. Subsequent flights or ground observations were obtained throughout the active season to monitor offspring survival or to fill in missing data for females not seen earlier due to poor observability (e.g., closed canopy cover). GPS and activity data were downloaded directly from collars collected from the field when bears either cast the collar or died. Our sample included the predicted‐parturient females from Roberts et al. (2026), excluding those assumed to be false positive detections of parturition events (i.e., one 2‐year‐old female and all females observed with yearlings or 2‐year‐old offspring). We categorized our sample of known‐ or predicted‐parturient females into three categories according to the apparent survival of their litter when first visually observed after den exit: successful (observed with cubs), unsuccessful (observed without cubs), and unknown status (not observed).

We calculated day of year for predicted parturition events (*Birth DOY*) from Roberts et al. (2026). Based on the estimated gestation period from previous studies [Tsubota et al. 1987 (60 days); Quest 2002 (54 days); Friebe et al. 2014 (56 days)], we estimated the date of implantation by subtracting 56 days from the predicted date of birth (*Implantation DOY*). For consistency with other values, *Birth DOY* was assigned values < 1 for the few parturition dates that occurred earlier than 1 Jan, and *Implantation DOY* was assigned values > 365 for the few observed implantation dates that occurred after 31 Dec.

We estimated dates of den entry (*ENTRY DOY*) and exit (*EXIT DOY*) using GPS‐ and VHF‐derived locations and associated activity data. Although transitions to and from hibernating physiology and cessation and onset of food intake are processes that can take place over days to weeks (Friebe et al. 2001; Reynolds et al. 1976; Judd et al. 1986; Van Daele et al. 1990), Evans et al. (2016) reported that denning behavior was tightly coupled with metabolic suppression, and studies have indicated that feeding activity is absent to minimal near dens (Nelson et al. 1983; Maeda 1985). By identifying clusters of locations associated with dens, our goal was to approximate the period when females were consuming few to no calories and undergoing the hibernation transitions. We identified den site GPS clusters and recorded the first and last days that females were within a radius of about 150 m from the center of the cluster. We then compared these dates with mean and median daily activity data for verification. If additional bouts of apparent denning behavior were indicated by the activity data and secondary GPS clusters lasting ≥ 7 days, we included those dates within the estimated denning period assuming females might have moved their den or hibernated in day beds close to the den site. Den site GPS clusters were unavailable for some females, either because contact with satellites was blocked while the bear was in the den, the GPS transmitter failed (but VHF continued working), or the bear entered and exited from the den during the period when the GPS transmitter was turned off for the winter. Denning dates for those individuals were estimated from activity and VHF data, first by identifying the timing of rapid change in activity and then by recognizing a mean daily activity value of approximately 20 (active seconds/10‐min interval) as an indicator of hibernation, based on bears with complete datasets. We calculated the number of days between the first date of denning and estimated birth (*Entry‐to‐Birth DAYS*) and between estimated birth and the last date of denning (*Birth‐to‐Exit DAYS*). We also recorded latitude of dens, either for the centroid of the GPS den cluster or for the GPS location closest to the denning period if no GPS cluster was available.

### 2.3 Hypothesis and Predictions

**FIGURE 1:**
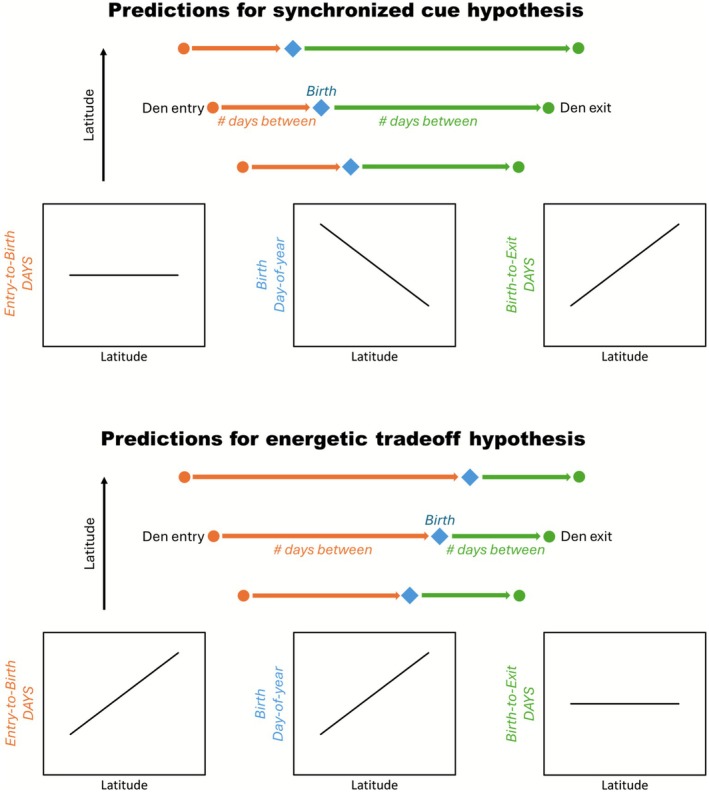
Graphical summary of predictions for two alternate hypotheses explaining the timing of parturition in grizzly bears.

We used general linear models (GLMs) and a one‐sample *t*‐test to evaluate predictions for our two hypotheses explaining the timing of parturition (Figure 1). In support of the synchronized cue hypothesis, we predicted *Implantation DOY* would coincide with *Entry DOY* (i.e., *Entry‐to‐Implantation DAYS* would not differ from 0). Consequently, *Entry‐to‐Birth DAYS* would be similar among females irrespective of latitude, *Birth DOY* would be negatively associated with latitude, and *Birth‐to‐exit DAYS* would be positively associated with latitude. In support of the energetic tradeoff hypothesis, we predicted *Birth‐to‐Exit DAYS* would be consistent among females irrespective of latitude. Consequently, *Entry‐to‐Birth DAYS* and *Birth DOY* would be positively associated with latitude, and *Entry‐to‐Implantation DAYS* would differ from 0.

Initial GLMs predicting *Birth DOY*, *Entry‐to‐Birth DAYS*, and *Birth‐to‐Exit DAYS* included latitude as a covariate, observation status (i.e., successful, unsuccessful, or unknown) as a factor, and an interaction term between latitude and observation status. We used this initial model structure to evaluate relationships separately for observation status categories, understanding that the occurrence and thus timing of parturition events was less certain for unsuccessful and unknown‐status females in our sample. If the interaction term was not supported (*p* > 0.05), we reran the GLM without the interaction term but retained observation status as a factor. We tested *Entry‐to‐Implantation DAYS* using a one‐sample *t*‐test with 0 as the critical value. Finally, we performed two sensitivity analyses. First, we repeated the analyses above while excluding GAAR females (i.e., the population with the most extreme values for latitude and the smallest sample size). Second, we repeated analyses twice with all unknown‐status females reassigned as successful and then unsuccessful.

## 3 Results

Our sample of predicted birth dates included 115 females known or predicted to have given birth: 57% successful (first observed with cubs), 22% unsuccessful (first observed without cubs), and 21% unknown (not observed; Table 1). Dates of first visual observation varied between 2 Apr and 12 Nov, with 52% of females observed within 2 weeks of den exit and 74% of females observed within 4 weeks of den exit (Table 2). As expected, *Entry DOY* was negatively associated with latitude (*F*_1,113_ = 34.35, *p* < 0.001, *β* = −1.34), while *Exit DOY* (*F*_1,102_ = 5.20, *p* = 0.025, *β* = 0.54) and duration of denning (*F*_1,102_ = 22.79, *p* < 0.001, *β* = 1.84) were positively associated with latitude (Table 2), providing the underlying framework necessary to test our predictions (Figure 1). Eleven females shed their collar before den exit, thus had missing values for *Exit DOY* and *Birth‐to‐Exit DAYS*. Among all females, *Birth DOY* ranged from 27 Dec to 28 Feb and *Implantation DOY* ranged from 1 Nov to 3 Jan (Table 2).

**TABLE 1:** Sample size of predicted births for female grizzly bears by study area and observation status: Successful (observed with cubs), unsuccessful (observed without cubs), and unknown (not observed).

Observation status	Greater Yellowstone Ecosystem	Northern Continental Divide Ecosystem	Selkirk and Cabinet‐Yaak Ecosystems	Gates of the Arctic Park and Preserve	Total
Successful	15	42	6	3	66
Unsuccessful	5	10	4	6	25
Unknown	3	12	5	4	24
Total	23	64	15	13	115

**TABLE 2:** Sample size, mean, and range for denning and parturition variables for female grizzly bears, by study area.

Variable	Measure	Greater Yellowstone Ecosystem	Northern Continental Divide Ecosystem	Selkirk and Cabinet‐Yaak Ecosystems	Gates of the Arctic Park and Preserve
Latitude	*N*	22	64	15	13
	Mean	44.19	48.20	48.92	67.27
	Range	43.35–45.15	47.03–48.96	48.58–49.53	67.03–67.64
First observation	*N*	20	52	9	8
	Mean	8 May	21 May	3 Jun	1 Jun
	Range	7 Apr–30 Jun	2 Apr–12 Nov	22 Apr–26 Jul	18 May–7 Jun
Entry DOY	*N*	23	64	15	13
	Mean	3 Nov	30 Oct	4 Nov	4 Oct
	Range	6 Oct–8 Dec	5 Oct–6 Dec	3 Oct–21 Dec	17 Sep–24 Oct
Exit DOY	*N*	22	57	15	10
	Mean	29 Apr	25 Apr	26 Apr	7 May
	Range	21 Mar–20 May	11 Mar–14 May	1 Apr–16 May	25 Apr–27 May
Denning duration	*N*	22	57	15	10
	Mean	177	177	174	216
	Range	103–205	122–220	125–215	188–245
Birth DOY	*N*	23	64	15	13
	Mean	10 Jan	20 Jan	28 Jan	7 Feb
	Range	27 Dec–23 Jan	31 Dec–24 Feb	13 Jan–16 Feb	16 Jan–28 Feb
Implantation DOY	*N*	23	64	15	13
	Mean	15 Nov	26 Nov	3 Dec	13 Dec
	Range	1 Nov–28 Nov	5 Nov–30 Dec	18 Nov–22 Dec	21 Nov–3 Jan

**FIGURE 2:**
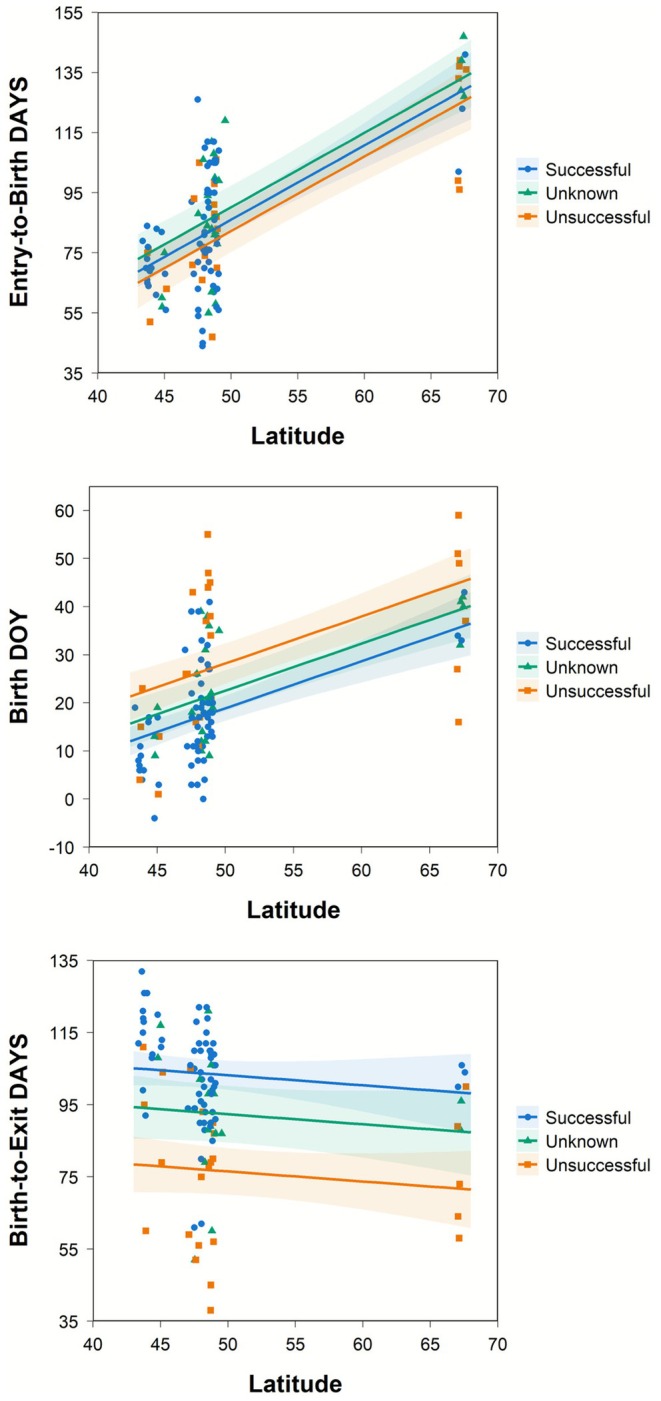
Observed values and fitted general linear models predicting grizzly bear number of days between den entry and birth (*Entry‐to‐Birth DAYS*; top), number of days between birth and den exit (*Birth‐to‐Exit DAYS*; center), and birth day of year (*Birth DOY*; bottom), as a function of latitude and observation status: Successful (observed with cubs), unsuccessful (observed without cubs), and unknown (not observed).

In the initial model predicting *Entry‐to‐Birth DAYS*, the interaction between latitude and observation status was not supported (*F*_2,1098_ = 0.26, *p* = 0.770) and this term was omitted. In the final model, latitude (*F*_1,111_ = 87.33, *p* < 0.001) was an important predictor, but observation status was not (*F*_2,111_ = 1.20, *p* = 0.306). The model predicted *Entry‐to‐Birth DAYS* increased 2.5 (95% CI: 1.9–3.0) days with each degree of latitude (Figure 2). This result supported the energetic tradeoff hypothesis.

In the initial model predicting *Birth DOY*, the interaction between latitude and observation status was not supported (*F*_2,109_ = 0.61, *p* = 0.548) and this term was omitted. In the final model, latitude (*F*_1,111_ = 39.16, *p* < 0.001) and observed female status (*F*_2,111_ = 6.75, *p* = 0.002) were both important predictors. The model predicted *Birth DOY* increased 1.0 day (95% CI: 0.7–1.3) with each degree of increasing latitude. This result supported the energetic tradeoff hypothesis. The model also predicted unsuccessful females (e.g., those that presumably lost their litter before observation) gave birth 9.3 days (95% CI: 4.3–14.4) later than successful females (*p* < 0.001).

In the initial model predicting *Birth‐to‐Exit DAYS*, the interaction between latitude and observation status was not supported (*F*_2,98_ = 0.22, *p* = 0.805) and this term was omitted. In the final model, latitude was not a predictor (*F*_1,100_ = 1.12, *p* = 0.293), supporting the energetic tradeoff hypothesis. Observation status was an important predictor (*F*_2,100_ = 23.29, *p* < 0.001). The model predicted that *Birth‐to‐Exit DAYS* was longest at 103.3 (95% CI: 99.1–107.4) days for successful females, while it was 26.7 days shorter (95% CI: 18.9–34.5) for unsuccessful females and 15.9 days shorter (95% CI: 5.6–26.1) for females with unknown status (*p* = 0.003).

*Implantation‐to‐Entry DAYS* differed from 0 (*t*_114_ = 13.08, *p* < 0.001), supporting the energetic tradeoff hypothesis. On average, *Implantation DOY* occurred 29.3 days (95% CI: 24.8–33.7) after *Entry DOY*. When we classified each predicted *Implantation DOY* as to whether it occurred before, during, or after the week of *Entry DOY* (i.e., ±3 days), we found proportions did not differ among populations (χ^2^_6_ = 2.67, *p* = 0.849, *n* = 115) or by observation status (χ^2^_4_ = 6.35, *p* = 0.175, *n* = 115). Overall, 5% of predicted implantation events occurred before the first week of denning (*Entry‐to‐Implantation DAYS* range: −32 to −4 days), 7% occurred during the first week of denning (*Entry‐to‐Implantation DAYS* range: −2 to 2 days), and 88% occurred after the first week of denning (*Entry‐to‐Implantation DAYS* range: 4–91 days).

In sensitivity models where we excluded observations from GAAR, tests for latitude were comparable to original models for *Entry‐to‐Birth DAYS* (*p* = 0.579), *Birth DOY* (*p* < 0.001, *β* = 2.90 days), and *Implantation‐to‐Entry DAYS* (*p* < 0.001, *β* = 25.5 days), supporting the energetic tradeoff hypothesis. In the model predicting *Birth‐to‐Exit DAYS*, latitude was a negative predictor (*p* < 0.001, *β* = −3.44 [95% CI: −1.73 to −5.16]), a result supporting neither hypothesis but more consistent with the energetic tradeoff hypothesis. In sensitivity analyses for observation status, all results were comparable to the original models. Observation status was not an important predictor for *Entry‐to‐Birth DAYS* when unknown‐status females were reassigned as successful (*p* = 0.229) or unsuccessful (*p* = 0.951). Predicted *Birth DOY* was 8.2 (*p* = 0.001) and 6.5 (*p* = 0.002) days earlier for successful versus unsuccessful females, when unknown‐status females were reassigned as successful and unsuccessful, respectively. Predicted *Birth‐to‐Exit DAYS* was 26.2 (*p* < 0.001) and 20.2 (*p* < 0.001) days longer for successful females versus unsuccessful females when unknown‐status females were reassigned as successful and unsuccessful, respectively.

## 4 Discussion

Our results, including sensitivity analyses, supported all predictions for the energetic tradeoff hypothesis (parturition timed with den exit) and none of the predictions for the synchronized cue hypothesis (parturition timed with den entry). This provided strong evidence that parturition is timed within the denning period to simultaneously minimize lactation costs for the mother while maximizing cub development time.

Whereas the energetic tradeoff hypothesis was strongly supported, we still observed wide variation in birth dates and the number of days between birth and den exit, and we offer two explanations. First, some of the variation is likely due to the stochastic nature of spring weather and food conditions (Evans et al. 2016; Pigeon et al. 2016; Delgado et al. 2018). Although optimal birth timing might be tied to the average spring conditions at a locality, possibly regulated by photoperiod (Folk et al. 1976), the realized timing of den exit and vegetation green‐up reflects annual and spatial stochasticity in spring temperature, moisture, and snowpack. For example, early versus late spring green‐up would cause some mismatch between optimal and realized timing. Indeed, this was suggested by our analyses, wherein *Birth DOY*, which we expect to be regulated by average conditions, varied within a 63‐day range, but *Birth‐to‐Exit DAYS*, which we expect to be influenced by concurrent conditions (Evans et al. 2016; Pigeon et al. 2016; Delgado et al. 2018), varied within a 94‐day range.

Second, given the hypothesized tradeoff is between the long‐term energetic welfare of the mother and survival potential of the current litter, variation in timing is entirely consistent with the hypothesis, whereby a population‐level, optimal‐timing strategy would be further modified by individual maternal body condition. Much of the observed variation in birth timing was explained by female observation status, providing some support for this concept. Although inclusion of observation status in our modeling was primarily driven by the need to account for known versus inferred births (i.e., potential false predictions of parturition events), the high predictive accuracy of the technique (Roberts et al. 2026) suggests most predictions were correct. Consequently, we deduced that observed differences between successful and other females have biological explanations. Variation in *Birth DOY* and *Birth‐to‐Exit DAYS* was smallest for the sample of successful females (i.e., those observed with cubs in the spring). Model‐predicted *Birth‐to‐Exit DAYS* averaged 103 days (SE: 2.0) for these successful females, a period that presumably optimized maternal energy balance and cub survival. *Birth‐to‐Exit DAYS* averaged only 77 days (SE: 3.3) among unsuccessful females (i.e., predicted parturient females that presumably lost their litter before the first observation in the spring) and the average for unknown‐status females was intermediate at 93 days (SE: 4.0), consistent with this sample likely being a mixture of successful and unsuccessful females that were not observed. One component accounting for these differences in *Birth‐to‐Exit DAYS* was an earlier average *Birth DOY* among successful females, which occurred about 9 days earlier than that of unsuccessful females. The larger component accounting for the differences in *Birth‐to‐Exit DAYS* was timing of den exit. On average, unsuccessful females emerged from their dens about 17 days earlier than successful females. Together, these results support the conclusion that later births or shorter pre‐emergence lactation periods resulted in cubs being at an earlier stage of development at den exit, which reduced cub survival and increased the risk of whole litter loss. It is also possible that pre‐emergence whole litter loss may have occurred, as inferred among Scandinavian brown bears (Zedrosser et al. 2009), providing an opportunity for unsuccessful mothers to exit their den at a time more similar to lone females or females with older offspring.

With such high costs to offspring survival, later births and earlier den emergence might appear to be maladaptive strategies. However, we propose that with a reproductive life that can span 20 years or more (Schwartz et al. 2003), a condition‐mediated strategy of timing birth relative to den exit would allow female grizzly bears to weigh their current reproductive effort against their potential long‐term fitness (e.g., Cole 1954; Drent and Daan 1980; Morano et al. 2013). Larger mothers, with greater lean body mass and fat reserves, would be able to shift birth timing earlier, thereby providing a longer period for their cubs to develop before den exit when spring green‐up is more advanced, with less risk to their own survival or future fitness. Females with fewer energetic reserves, particularly inexperienced mothers with potentially lower prospects for offspring survival (e.g., Hadley et al. 2007; Engebretsen et al. 2024), would benefit from a strategy that minimizes the pre‐emergence lactation period, thus preserving resources for future reproduction, even at the cost of the current litter. However, as opposed to abandoning a pregnancy altogether by reabsorbing the blastocyst before implantation (a hypothesized but unverified strategy, e.g., Rogers 1976, Bunnell and Tait 1981, Robbins et al. 2012), adjustment of birth timing would allow poorer condition females to safeguard their own survival potential while still investing some energy toward a current reproductive effort. A physiological mechanism facilitating this strategy would be adaptive provided there was some probability that cubs at an earlier stage of development would survive, possibly contingent on favorable spring conditions and early resumption of foraging. Our data confirmed that spring and even first‐year survival of cubs that emerged at a younger age was possible, as several successful females in our sample that emerged 61–90 days after birth were observed with their litter throughout the cub year.

Our energetic tradeoff hypothesis and associated findings were supported by several previous studies that recognized the pertinent energetic considerations we examined here. For example, several studies of American black bears and grizzly bears have reported that older or larger females gave birth earlier than younger or smaller females (Bridges et al. 2011; Robbins et al. 2012; Mesa Cruz 2018; Lemière et al. 2022). In these previous studies, authors seemed to presume a fixed Jan–Feb parturition time frame and characterized observed variation as stemming from proximate mechanisms or constraints on younger or leaner females. Robbins et al. (2012) surmised that females in poorer condition produced offspring at a reduced cost by shortening lactation time during hibernation. López‐Alfaro et al. (2013) suggested that, within a population, variation in birth timing might be the primary mechanism used to adapt to inter‐annual food availability. Lemière et al. (2022) reported females in a higher‐latitude population gave birth later than those in a lower‐latitude population and suggested that later parturition in the northern population might ensure that females do not compromise their own survival while simultaneously nursing and fasting. Importantly, because few late‐born litters survived, Lemière et al. (2022) argued later parturition was simply a consequence of insufficient fat stores and not an energetic strategy. Our results, however, emphasize that the two opposing energetic demands (i.e., minimizing the in‐den lactation time and maximizing cub developmental time) impact all pregnant females, not just those in poorer condition or those in more northern latitudes. Ultimately, the necessary balancing of this energetic tradeoff represents a reasonably flexible evolutionary strategy that explains birth timing across all populations.

Although body condition measurements were collected at the time of capture, females generally wore GPS‐collars for several years; therefore, we had only limited data to directly test a body‐condition effect on birth timing. However, we were able to evaluate possible age effects in post hoc analyses, although these results should not be considered definitive because age is not a direct indicator of body condition. We found that 44% of the unsuccessful sample were growing‐age females (≤ 6 years old; refer to Corradini et al. 2023), compared with only 20% of the successful sample and 25% of the unknown‐status females (χ^2^_2_ = 5.57, *p* = 0.062). In models including latitude, age (*F*_1,101_ = 10.00, *p* = 0.002, *β* = 1.09), and age class (*F*_1,103_ = 12.07, *p* < 0.001, *β* = 14.35) were important predictors of *Birth‐to‐Exit DAYS*; however, they were not predictors of *Birth DOY* (*p* = 0.583).

López‐Alfaro et al. (2013) modeled the energetic costs of brown bear reproduction to determine how maternal condition, length of lactation, litter size, and length of hibernation affect success, and their assessment involved a simulated pre‐exit lactation period of 60–74 days. They recognized this range probably underestimated the length of lactation for many wild bears in northern latitudes. Our results showed that the actual pre‐exit lactation period often exceeded 100 days at all latitudes, as did Lemière et al. (2022), who reported that cubs were ≥ 105 days old at den exit. A 74‐day period was more consistent with the *Birth‐to‐Exit DAYS* observed for unsuccessful females than for successful females.

Our results provide strong evidence for independence between den entry and implantation cues, confirming earlier interpretations (Palmer et al. 1988, Hellgren et al. 1998) and more recent evidence (Friebe et al. 2014; Lemière et al. 2022). However, we note that average denning duration and optimal *Birth‐to‐Exit DAYS* would be expected to align in some regions to create similar timing for den entry and implantation, perhaps helping to explain some instances of coincidence of these behaviors (e.g., Mesa Cruz 2018). If we assume that 103 days for *Birth‐to‐Exit DAYS* (i.e., the mean for successful females in our study) approximates optimal timing and add a 56‐day gestation period, den entry and implantation would coincide for grizzly bears when denning duration is roughly 159 days. In a review of brown bear denning dates worldwide, Krofel et al. (2017) reported female denning duration nearest this value for populations in the GYE and Alberta, Canada. This matches with our analyses that predicted that *Entry‐to‐Birth DAYS* was about 56 for successful bears near 44 degrees latitude in interior North America, corresponding to the GYE (Figure 2, top). As most of our study bears were at higher latitudes, we found predicted implantation occurred after den entry for the majority of females, by as many as 91 days. Most populations summarized by Krofel et al. (2017) also denned for periods exceeding 159 days, but females in two populations (Greece and Slovenia) denned ≤ 107 days. Our hypothesis and results suggest implantation among these females would occur well before den entry. It would appear then that the start of gestation is not a driver for females to enter their dens (e.g., Tsubota et al. 1990; Hissa 1997), so an alternative explanation is needed for the earlier den entry of pregnant females (Servheen and Klaver 1983; Judd et al. 1986; Van Daele et al. 1990; Friebe et al. 2001; Pigeon et al. 2016). Perhaps pregnant females are risk‐averse to losing fat stores by continuing to stay active during a period of dwindling food supply, whereas non‐pregnant females accept more risk, particularly if potential windfall food sources may be available (e.g., high‐energy ungulate remains left by hunters; Haroldson et al. 2004). It might also be reasonable to expect pregnant females, unaccompanied by dependent offspring, are more efficient foragers and build up fat stores quicker during hyperphagia compared with current mothers, who often allocate some of their energy toward lactation and might have reduced food intake because they share food patches with their dependent offspring.

Bridges et al. (2011) postulated that later parturition by younger, smaller female American black bears may be advantageous by allowing them to delay den exit to a time when more food resources would be available. We found that *Exit DOY* was positively, not negatively, associated with bear age (*F*_1,101_ = 17.22, *p* < 0.001, *β* = 1.07), contrary to this idea. Nevertheless, delaying den exit to a time when more food is available may be a viable explanation for the later den emergence of all females with cubs (e.g., McLoughlin et al. 2002; Graham and Stenhouse 2014; González‐Bernardo et al. 2020).

Whereas our results provide strong evidence for the energetic‐tradeoff hypothesis for birth timing, we acknowledge the occurrence and timing of birth events analyzed here were inferred from accelerometer data (Roberts et al. 2026). Using our technique or other methods for documenting birth dates (e.g., cameras, microphones), other researchers could corroborate our birth timing findings with brown bears in other regions or with other bear species in the Ursinae subfamily. In particular, we note that our conceptual framework linking birth timing with den emergence also appears consistent with the obligate maternal denning of polar bears ( *Ursus maritimus* ), which commences in the fall after an abbreviated seal (Phocidae) foraging season compared with other sex or age categories (Stirling et al. 1977; Derocher and Stirling 1990); ends coincident with increasing prey availability due to pupping and molting season for seals (Stirling and Archibald 1977; Smith 1980); and requires time for cubs to develop sufficiently to travel the sea ice and minimize heat loss in frigid arctic temperatures (Blix and Lentfer 1979; Rode et al. 2018).

### 4.1 Management Implications

Several studies (e.g., Pigeon et al. 2016; González‐Bernardo et al. 2020; Lemière et al. 2022) have postulated that climate change causes a temporal mismatch between birth and den emergence leading to dramatic reproductive consequences, whereby warming will compel parturient females to exit dens too early when their offspring are small and vulnerable to mortality. The energetic tradeoff hypothesis suggests that females might be resilient to climate change. A flexible birth‐timing strategy would be evolutionarily favored in a species with highly variable food economies and a currently wide and historically wider distribution, such as grizzly bears. As bears display plasticity in matching their denning chronology to a wide array of local food and climate conditions (Fowler et al. 2019), females may be similarly capable of matching their birth timing to this variation. We do not know the mechanism(s) by which individual bears might make such advantageous adjustments to birth timing, but wide variation in birth dates we observed among and within populations suggests the mechanism is likely influenced by current, local conditions. Our results suggest that, over a wide range of latitudes, natural selection has favored a relatively consistent *Birth‐to‐Exit Days* under average body conditions, along with shifts toward later or earlier births for females with lower or higher levels of bodily stored energy, respectively. Instead of considering later shifting of birth timing simply as an unavoidable constraint, we propose it is another example of adaptive allocation of energetic resources by bears. This inherent strategy of optimizing resource allocation to balance current and lifetime fitness is consistent with other adaptations, such as how bears allocate food energy toward body fat versus lean body mass (Corradini et al. 2023), an adaptation that likely explains wide variation in asymptotic body size across habitats with varying food abundances (e.g., Hilderbrand et al. 2019).

Finally, our study provides important context for understanding relationships between food access and population dynamics. Lemière et al. (2022) suggested that later birth timing could become more frequent as a result of climate‐induced food limitation, such as potential reduction of bilberry ( *Vaccinium myrtillus* ) production in Scandinavia. This effect would be consistent with our hypothesis, at least in the short‐term. Additionally, evidence for intrinsic density‐dependent effects on vital rates has begun to emerge. Consistent with their long‐lived life history strategy (Eberhardt 1977), the demographic rates observed to change the most with increasing population density have been cub and yearling survival, age of first reproduction, and litter interval (Ciarniello et al. 2009; Zedrosser et al. 2006; McLellan 2015; van Manen et al. 2016; Keay et al. 2018). A leading mechanism proposed for reduced cub survival in high‐density brown bear populations has been intraspecific killing (van Manen et al. 2016) or more specifically sexually selected infanticide (Swenson et al. 1997). Our results offer an alternative, indirect mechanism for explaining reduced cub survival at higher population densities (i.e., higher frequency of cubs at an earlier developmental stage at den exit), potentially linked with females' diminished acquisition of food energy caused by intraspecific interference competition (Corradini et al. 2023). In turn, it might also suggest a possible mechanism for the higher ages of first reproduction and longer birth intervals observed among some higher‐density populations (Ciarniello et al. 2009; McLellan 2015; Keay et al. 2018). This energetic, birth‐timing explanation for declining population growth rates at higher densities may be an important consideration in making management decisions, particularly if male‐biased hunter harvest is presumed to improve cub survival through reduced infanticide.

## Author Contributions

**Cecily M. Costello:** conceptualization (lead), data curation (equal), formal analysis (lead), methodology (lead), writing – original draft (lead), writing – review and editing (lead). **Lori L. Roberts:** conceptualization (lead), data curation (equal), formal analysis (lead), methodology (lead), writing – original draft (supporting), writing – review and editing (supporting). **Daniel D. Bjornlie:** data curation (equal), formal analysis (supporting), methodology (supporting), writing – review and editing (supporting). **Matthew D. Cameron:** data curation (equal), formal analysis (supporting), methodology (supporting), writing – review and editing (supporting). **Justin G. Clapp:** data curation (equal), formal analysis (supporting), methodology (supporting), writing – review and editing (supporting). **Mark A. Haroldson:** data curation (equal), formal analysis (supporting), methodology (supporting), writing – review and editing (supporting). **Grant V. Hilderbrand:** data curation (equal), formal analysis (supporting), methodology (supporting), writing – review and editing (supporting). **Kyle Joly:** data curation (equal), formal analysis (supporting), methodology (supporting), writing – review and editing (supporting). **Wayne F. Kasworm:** data curation (equal), formal analysis (supporting), methodology (supporting), writing – review and editing (supporting). **Jeremy M. Nicholson:** data curation (equal). **Thomas G. Radandt:** data curation (equal). **Mathew S. Sorum:** data curation (equal), formal analysis (supporting), methodology (supporting), writing – review and editing (supporting). **Justin E. Teisberg:** data curation (equal), formal analysis (supporting), methodology (supporting), writing – review and editing (supporting). **Frank T. van Manen:** data curation (equal), formal analysis (supporting), methodology (supporting), writing – review and editing (supporting). **Milan A. Vinks:** data curation (equal), formal analysis (supporting), methodology (supporting), writing – review and editing (supporting).

## Funding

This work was supported by U.S. Forest Service; National Park Service; U.S. Fish and Wildlife Service; Montana Fish, Wildlife and Parks; Wyoming Game and Fish Department; U.S. Geological Survey, Rocky Mountain Science Center, Interagency Grizzly Bear Study Team; Idaho Fish and Game Department; Blackfeet Fish and Wildlife Department; and Confederated Salish and Kootenai Tribal Natural Resources Department.

## Conflicts of Interest

The authors declare no conflicts of interest.

## Data Availability

The data used for this study are available online in Costello et al. (2025) https://doi.org/10.5066/P14WTKOF.
